# Dyadic body competence predicts movement synchrony during the mirror game

**DOI:** 10.3389/fnhum.2024.1401494

**Published:** 2024-06-19

**Authors:** Ryssa Moffat, Leonie Roos, Courtney Casale, Emily S. Cross

**Affiliations:** ^1^Professorship for Social Brain Sciences, ETH, Zurich, Switzerland; ^2^Institute of Cognitive Science, Osnabrück University, Osnabrück, Germany; ^3^School of Psychological Sciences, Macquarie University, Sydney, NSW, Australia

**Keywords:** embodiment, motor synchrony, mirror game, body competence, body perception, kinematics, perception-action coupling

## Abstract

The process of synchronizing our body movements with others is known to enhance rapport, affect, and prosociality. Furthermore, emerging evidence suggests that synchronizing activities may enhance cognitive performance. Unknown, by contrast, is the extent to which people’s individual traits and experiences influence their ability to achieve and maintain movement synchrony with another person, which is key for unlocking the social and affective benefits of movement synchrony. Here, we take a dyad-centered approach to gain a deeper understanding of the role of embodiment in achieving and maintaining movement synchrony. Using existing data, we explored the relationship between body competence and body perception scores at the level of the dyad, and the dyad’s movement synchrony and complexity while playing a 2.5-min movement mirroring game. The data revealed that dyadic body competence scores positively correlate with movement synchrony, but not complexity, and that dyadic body perception scores are not associated with movement synchrony or complexity. Movement synchrony was greater when the more experienced member of the dyad was responsible for copying movements. Finally, movement synchrony and complexity were stable across the duration of the mirror game. These findings show that movement synchrony is sensitive to the composition of the dyad involved, specifically the dyad’s embodiment, illuminating the value of dyadic approaches to understanding body movements in social contexts.

## 1 Introduction

As social beings, humans synchronize their behaviors with those exhibited by other humans (Lakin et al., 2003; Rennung and Göritz, 2016; Vicaria and Dickens, 2016). Humans match each other’s limb and trunk movements, facial expressions, emotional reactions, and even vocal patterns (Borrie and Delfino, 2017; Lahnakoski et al., 2020; Yokozuka et al., 2021; Shamay-Tsoory, 2022). Inside bodies, involuntary synchrony can emerge between the hearts, endocrine systems, and brains of the self and other (Feldman, 2017; Nummenmaa et al., 2018; Parkinson et al., 2018; Mayo and Gordon, 2020; De Hamilton, 2021). Generally speaking, synchrony between people is believed to support social learning and social alignment (Bandura and Adams, 1977; Fuhrmann et al., 2014; Shamay-Tsoory et al., 2019; Crone et al., 2021; Pan et al., 2021) which can, in turn, strengthen social bonds (Lakin et al., 2003). The social benefits of *movement synchrony*, i.e., matching other people’s body movements, have been investigated in depth, and the rather colloquial consensus is that movement synchrony functions as a form of ‘social glue’ (Lakin et al., 2003). This consensus is built on a wealth of studies demonstrating that movement synchrony fosters feelings of social closeness or togetherness, positive affect, and prosociality (Chartrand and Lakin, 2013; Rennung and Göritz, 2016; Vicaria and Dickens, 2016; Mogan et al., 2017; Zampella et al., 2020). Additional evidence suggests that inducing movement synchrony through synchronizing activities, such as the mirror game, can be operationalized to enhance cognitive performance (Keisari et al., 2022; Moffat et al., 2024).

The level of movement synchrony between two people playing the mirror game varies substantially across pairs of people (dyads, henceforth; Noy et al., 2011; Ravreby et al., 2022; Moffat et al., 2024). From a logical standpoint, this variability is to be expected, given that dyads are composed of unique individuals with their own personality traits, social experiences and bodily competencies. In terms of the consequences of such individual differences for social interactions, dyads who achieve greater movement synchrony during the mirror game are more likely to experience positive affect (Tschacher et al., 2014), ‘like’ each other (Ravreby et al., 2022) and enjoy the interaction more (Lahnakoski et al., 2020) compared to dyads whose movements are less synchronous. Moreover, the probability of dyads liking each other has been demonstrated to be closely linked to the complexity and novelty of the movements produced during the mirror game: Greater synchrony and liking of one’s partner ensued from mirror-game movements that were more complex and novel (Ravreby et al., 2022). Ravreby et al.’s (2022) work provides initial insights into the kinematic features underpinning the development of social bonds and takes important steps in considering liking from a mutual, *dyadic*, perspective (i.e., using ratings of liking averaged across dyads). This work represents a positive start to exploring dyadic perspectives for examining the emergence and maintenance of movement synchrony. Dyadic measures, which have been overlooked to date, give context about the makeup of the dyad *per se*. For example, dyadic measures of personality traits, and measures of bodily experiences/expertise that capture dyad-level differences in embodiment may be particularly informative for understanding the emergence and maintenance of movement synchrony.

With respect to personality traits, numerous studies examine how dyadic measures of personality traits relate to social behaviors, such as prosociality and relationship quality (e.g., Heerey, 2015; Bolis et al., 2021). In contrast, only a handful of studies examine the link between dyadic measures of personality traits and body movements during social interactions between two humans (Carlson et al., 2019; Lahnakoski et al., 2020; Arellano-Véliz et al., 2023) and also between humans and non-human agents (i.e., robots; Aly and Tapus, 2013). Arellano-Véliz et al. (2023) recently demonstrated that dyadic measures of openness and agreeability may predict the degree of movement synchrony during conversation. Specifically, the authors found that greater agreeableness and extraversion within dyads contributed to increased movement synchrony, and greater agreeableness also contributed to increased movement complexity. Carlson et al. (2019) measured dancing dyads’ trait empathy and found that dyadic levels of empathy were not related to phase locking of dance movements. The authors did, however, demonstrate that observers perceived dance movements performed by dyads composed of two individuals with high empathy scores as less similar than movements performed by dyads comprising one or more individuals with a low empathy score (Carlson et al., 2019). These findings demonstrate the relevance of dyad-level differences for gaining insight into the emergence of movement synchrony. Given that very few studies, to our knowledge, have taken this approach, further exploration into the makeup of dyads is necessary to continue developing our understanding of how and why movement synchronizing activities, such as those performed during the mirror game, offer social benefits.

Beyond personality traits and ratings of ‘liking’ one’s synchronized partner, we propose that individual differences in embodiment are likely to help explain the emergence of movement synchrony, and perhaps movement complexity, in synchronizing activities like the mirror game. We base this proposal on research showing that expert movement improvisors and trained dancers, i.e., individuals with very high levels of embodiment acquired through intensive and prolonged training, are able to perceive subtle details in movements which allow them to synchronize with others with ease (Noy et al., 2011; Honisch, 2012; Hart et al., 2014; Kirsch et al., 2016). Congruent evidence from brain imaging studies suggests that greater embodiment (expertise) results in greater activation of a network of brain regions believed to support the simulation of observed actions (Cross et al., 2006; Jola et al., 2012; Kirsch et al., 2016; Rizzolatti and Sinigaglia, 2016). Moreover, embodiment shapes the way members of the general population engage in social interactions, potentially involving movement synchrony (Garrod and Pickering, 2009; Quesque and Coello, 2015) and appreciating synchrony when observing dyadic movements (Moffat and Cross, 2024). Considering the evidence from expert movers and the general public, it stands to reason that the level of bodily experience or expertise possessed by members of a dyad might shape their ability to achieve and maintain synchrony, as well as the complexity of their movements while doing so.

In the present study, we focus on the extent to which dyadic levels of embodiment predict movement synchrony and complexity in the mirror game. More specifically, we explore how dyadic measures of beliefs regarding one’s ability to complete physical activities (e.g., body competence; Miller et al., 1981) and sensitivity to internal bodily signals (e.g., body perception; Cabrera et al., 2018) are related to movement synchrony and complexity during the mirror game. Measures of body competence and body perception capture embodiment from opposing perspectives. Body competence scores offer a subjective index of one’s cumulative experience engaging in physical activity, which is likely to correlate positively with levels of movement synchrony and complexity. Greater body perception scores are suggested to reflect an inward form of embodiment, which may detract from perception of subtle body movements performed by others and social interactions more generally (Wiebking et al., 2010; Lumsden et al., 2012; Ciaunica et al., 2023; Critchley et al., 2023; Moffat and Cross, 2024). That is, greater attention to internal bodily signals may be associated with reduced levels of synchrony (Lumsden et al., 2012) and movement complexity while participating in a synchronizing activity (Arellano-Véliz et al., 2023). A deeper understanding of the role of embodiment in achieving and maintaining movement synchrony holds implications for understanding the emergence and maintenance of spontaneous movement synchrony in collaborative daily activities (i.e., passing items over a barrier), performing arts (i.e., learning dance choreography), leisure activities (i.e., in a yoga class, or during contact improvisation), and potentially, interactions with non-human agents (i.e., social robots for companionship).

We set out to explore the extent to which dyadic levels of embodiment (i.e., body perception and competence scores averaged within dyads) are associated with the synchrony and complexity of dyads’ movements across the course of a mirror game. We preregistered the following exploratory hypotheses on the Open Science Framework:^1^ Movement synchrony will be positively correlated with dyadic body competence scores and negatively correlated with dyadic body perception scores. Movement complexity will be negatively correlated with dyadic body competence scores and positively correlated with dyadic body perception scores. Further, movement synchrony and complexity may change linearly over the course of the mirror game, and this trajectory may interact with dyadic measures of body competence and/or body perception.

## 2 Methods

### 2.1 Participants

We used data from 28 participants (12 female, 16 male, mean age 21.14 ± 5.08 years) that were collected for a previous study, where participants played the mirror game with an experimental confederate before completing a cognitive task paired with brain imaging (Moffat et al., 2024). Participants were recruited from Macquarie University in Sydney, Australia. All participants met the self-reported inclusion criteria of being right-handed, aged 18–40, having no history of head injury, neurological or psychiatric diagnoses, and not currently taking a psychopharmaceutical medication (SSRIs or Ritalin). Additionally, as the data were collected in the framework of a larger neuroimaging study, participants were included if they reported no alcohol consumption within the 12 h prior to the study or tetrahydrocannabinol (THC) use/exposure within the 24 h prior to the study, and not playing video games frequently (e.g., more than once a week). These were the inclusion criteria for the neuroimaging study from which these secondary data originate, wherein cognitive performance and brain activity during a response inhibition task were measured (Moffat et al., 2024). In this previous study, we applied the same criteria as an existing study with a similar design (König et al., 2021).

Participants were matched with one of two confederates; A: female, age 21; B: female, age 30; see Moffat et al. (2024) for description of counterbalancing. The confederate was introduced as a new student volunteer visiting the lab for the first time (i.e., a peer to the participant, who was equally as unfamiliar with the experiment and the experimenter). Of the 28 participants, 14 were matched with confederate A (6 female, 8 male, mean age 20.93 ± 5.98 years) and the other 14 with confederate B (6 female, 8 male, mean age 21.36 ± 4.2 years).

Ethical approval for the original study by Moffat et al. (2024) was obtained from the Macquarie University Human Research Ethics Committee (Ref: 520221102239451). Written consent was obtained from participants before the start of the experiment. Each participant received either course credit or cash honorarium (AUD $30) for their participation.

### 2.2 Measures of embodiment

Participants completed two self-report questionnaires, which we employ here as proxies for embodiment. The first, the Body Competence Questionnaire (BCQ; Miller et al., 1981) assesses beliefs about one’s body’s ability to complete physical activities. The BCQ employs a 5-point Likert scale (0 = *extremely uncharacteristic* and 4 = *extremely characteristic*) with statements like ‘For my size, I’m pretty strong’ or ‘I’m capable of moving quickly’. The second, the Body Perception Questionnaire Short Form (BPQ SF; Cabrera et al., 2018), assesses the degree to which an individual attends to their internal bodily signals. This scale also employs a 5-point Likert scale (1 = *never* and 5 = *always*) with statements such as ‘I notice myself swallowing frequently’, ‘I notice my mouth being dry’. We then averaged each of these measures separately per dyad, to obtain a joint measure per dyad, and subsequently z-scored the values at the group level.

### 2.3 Mirror-game procedure

**Figure 1:**
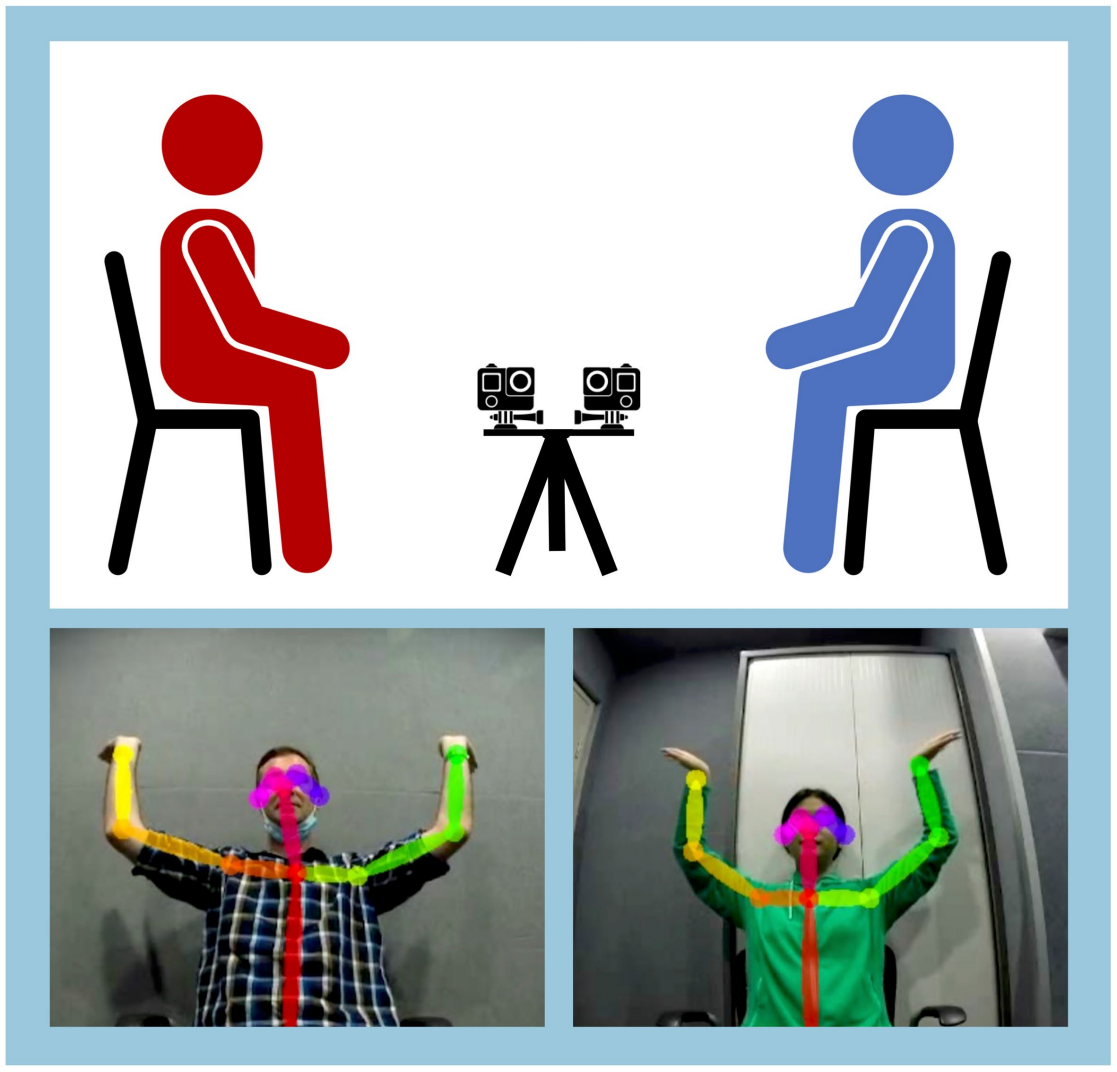
Top: Participant (red) plays the mirror game with an experimental confederate (blue). Upper-body movements were video-recorded using a pair of GoPros, one facing each member of the dyad. Bottom: Movement synchrony and complexity were calculated using the coordinates of each person’s joints per frame, as tracked using OpenPose software.

All dyads played the mirror game for a total of 5 min. To do so, the dyad (participant and confederate) sat facing one another (Figure 1). Each member took a 2.5-min turn leading the game, i.e., moving their arms spontaneously, while the other member matched their arm movements as closely as possible in space and time (Feniger-Schaal et al., 2021; Ravreby et al., 2022). The participant always led the first 2.5 min, followed by the confederate leading the second 2.5 min. This was done to allow individual participants to move spontaneously without any bias from having observed an example performed by the confederate. Dyads’ arm movements were video-recorded using two GoPro video cameras positioned on a tripod between the participants. The cameras were positioned slightly above knee height as not to obstruct the dyad’s eye contact (Figure 1).

### 2.4 Calculation of movement synchrony and complexity

We divided the 2.5-min videos (*N* = 56, 2 per dyad, i.e., participant leading and participant following) into 10 15-s segments.

#### 2.4.1 Movement synchrony

As described in Moffat et al. (2024), the dyad’s mean pose similarity was obtained using estimated x and y coordinates per joint obtained from OpenPose software (Cao et al., 2021). We used Moffat et al.’s (2024) R code, which was previously adapted from Broadwell and Tangherlini (2021), to estimate the Euclidean distance between all pairs of body parts for each person in a frame [i.e., calculating the distances between each combination of body parts (*n* = 42) for the participant]. These distances were stored in a separate ‘pose matrix’ per person and these pose matrices were then compared (via Laplacian procedure) for each frame to establish similarity per frame. The comparison returned estimates of movement synchrony between 0 = *no similarity* and 1 = *identical*. We multiplied these by 100, for comparability to Moffat et al. (2024), and calculated the mean per dyad across the 15-s segment of the video. Finally, we converted the values to z-scores to homogenize the scale with that of the embodiment measures.

#### 2.4.2 Movement complexity

We used sample entropy (Shannon and Weaver, 1949) as a proxy for movement complexity. In research investigating the kinematics of human movement, the term ‘complexity’ is used interchangeably with ‘predictability’ and ‘entropy’ in the literature, to describe sample entropy (Orlandi et al., 2020; Ravreby et al., 2022; Moffat and Cross, 2024). We computed the sample entropy of the x- and y-coordinates of left and right wrists of both members of a dyad across each 15-s segment of each video using the R package pracma (version 2.4.2; Borchers, 2022). Next, we averaged these values per 15-s segment. Entropy values closer to zero indicate lower complexity of the movements, whereas higher entropy values reflect increased complexity. Henceforth, we refer only to movement complexity.

### 2.5 Data analysis

To explore the influence of embodiment at the level of the dyad on movement synchrony and complexity in this secondary dataset, we used the lme4 package (version 1.1–34; Bates et al., 2015) in R (version 4.3.1; RStudio Team, 2023) within the RStudio IDE (version 2023.06.1; R Core Team, 2023). As per our preregistration, we built two linear mixed effects models, one focusing on movement synchrony, the other on movement complexity. We added parameters to the model incrementally, beginning with varying intercepts per participant and for the index of session per confederate, to account for potential incremental increases in expertise accrued by the confederate from one experimental session to the next. Subsequently, we added the simple parameters of dyadic body competence and dyadic body perception scores, followed by time (i.e., the index of the 15-s window in the 2.5-min recording of the mirror game). Next, we added separate 2-way interactions between time and each dyadic body competence and dyadic body perception separately.

Parameters were retained if they contributed substantially to explaining the variance in the data (Bates et al., 2015). We preregistered that we would include the index of confederates’ sessions as a varying intercept to account for confederates’ incremental acquisition of expertise across sessions. Ultimately this variable increased the complexity of our models to a degree that rendered the parameter estimates unreliable (i.e., singular or 0 convergence warning from the lme4 R package). Thus, we excluded the index of confederates’ sessions from the models and took a simpler approach to account for the confederates’ overall expertise: We included the role of the participant as ‘leader’ or ‘follower’ in the mirror game. If the participant was the leader, this meant the confederate was the follower, and we could thus partial out and interpret how the confederates’ expertise might influence movement synchrony. The final model predicting movement synchrony was *MovementSimilarity ~ 1 + BodyCompetence + BodyPerception + Role + (1|ID)*. The final model predicting movement complexity was *MovementComplexity ~ 1 + BodyCompetence + BodyPerception + Role + (1|ID)*. Time was not included as a parameter in our final models, as it did not explain substantial variance in the data.

A note regarding statistical power – these analyses and the associated hypotheses were explicitly preregistered as ‘exploratory’. We present our findings as starting points for hypothesis generation, to be examined in future studies with greater statistical power.

## 3 Results

**Figure 2:**
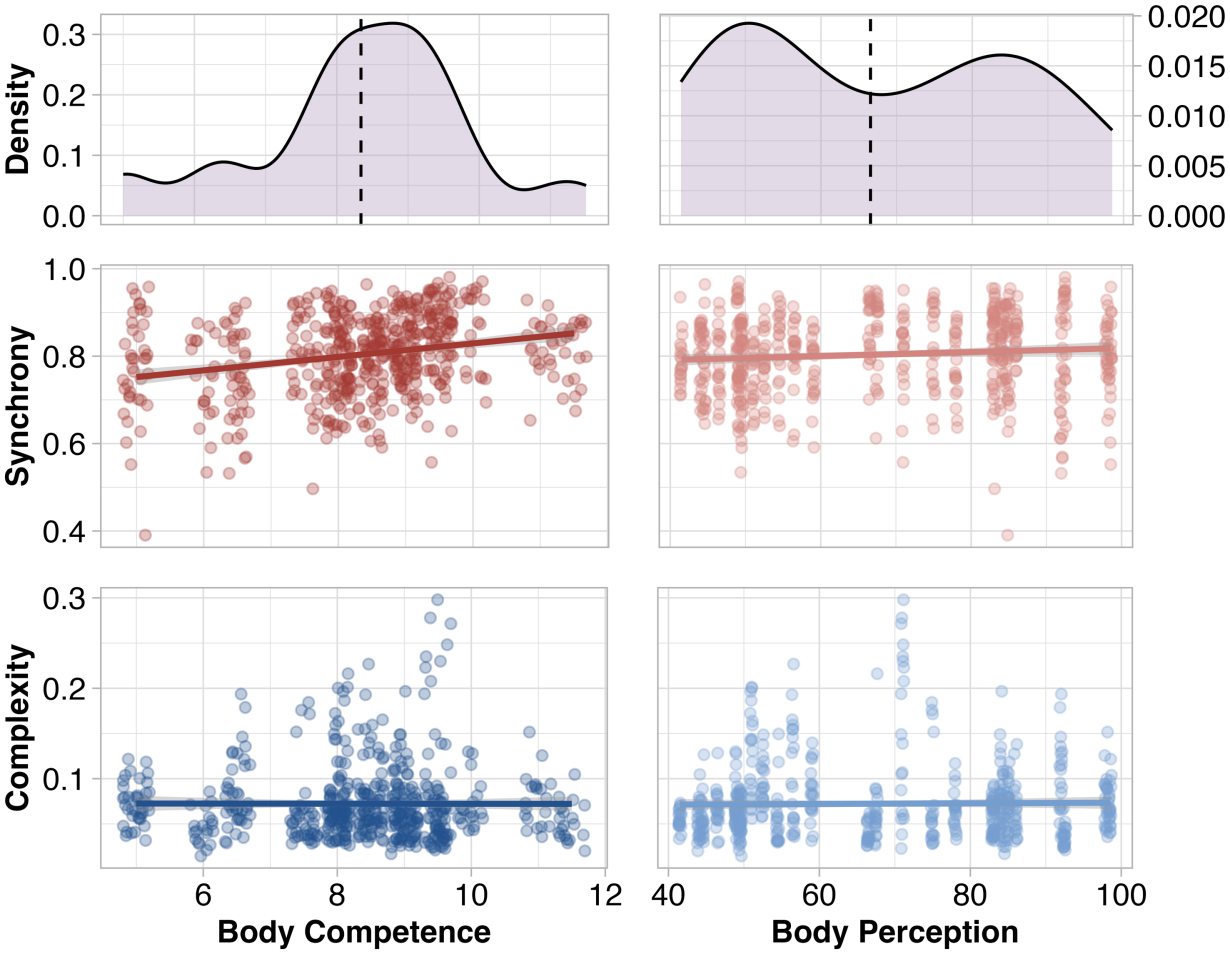
Top row: Distribution of dyadic body competence and body perception scores. Dashed line indicates the mean. Middle row: Relationship between movement synchrony and dyadic measures of body competence and body perception. Bottom row: Relationship between movement complexity and dyadic measures of body competence and body perception.

During the mirror game, mirroring resulted in a high degree of movement synchrony (mean = 0.80, SD = 0.09; possible range = 0–1). The movement complexity ranged from 0.01 to 0.30 (mean = 0.07, SD = 0.04; closer to zero signifies lower complexity). Dyadic body competence scores showed a relatively normal distribution, as both confederates had similar individual body competence scores (mean = 8.34, SD = 1.54; possible range = 0–16; top left panel in Figure 2). Dyadic body perception scores showed a weak bimodal distribution, resulting from the two confederates having substantially different individual body perceptions scores (mean = 66.55, SD = 18.64; possible range = 26–130; top right panel in Figure 2).

Next, we set out to explore the relationships between movement synchrony and complexity during the mirror game and two measures of dyadic embodiment, i.e., body competence and body perception. We hypothesized that movement synchrony would be positively associated with dyadic body competence and negatively associated with dyadic body perception. The data confirmed the expected positive association with body competence [*ß* = 0.02, SE = 0.01, 95% CI (0.01, 0.04), *p* < 0.001], but showed no evidence for the expected negative association with body perception [*ß* = 0.01, SE = 0.01, 95% CI (−0.002, 0.02), *p* = 0.081; middle row of Figure 2].

Next, we anticipated that movement complexity would be positively associated with body competence and negatively associated with body perception. We found no evidence to support either of these hypothesized relationships [body competence: *ß* = 0.00, SE = 0.01, 95% CI (−0.10, 0.10), *p* = 0.901; body perception: *ß* = 0.00, SE = 0.01, 95% CI (0.10, 0.10), *p* = 0.996; bottom row of Figure 2].

**Figure 3:**
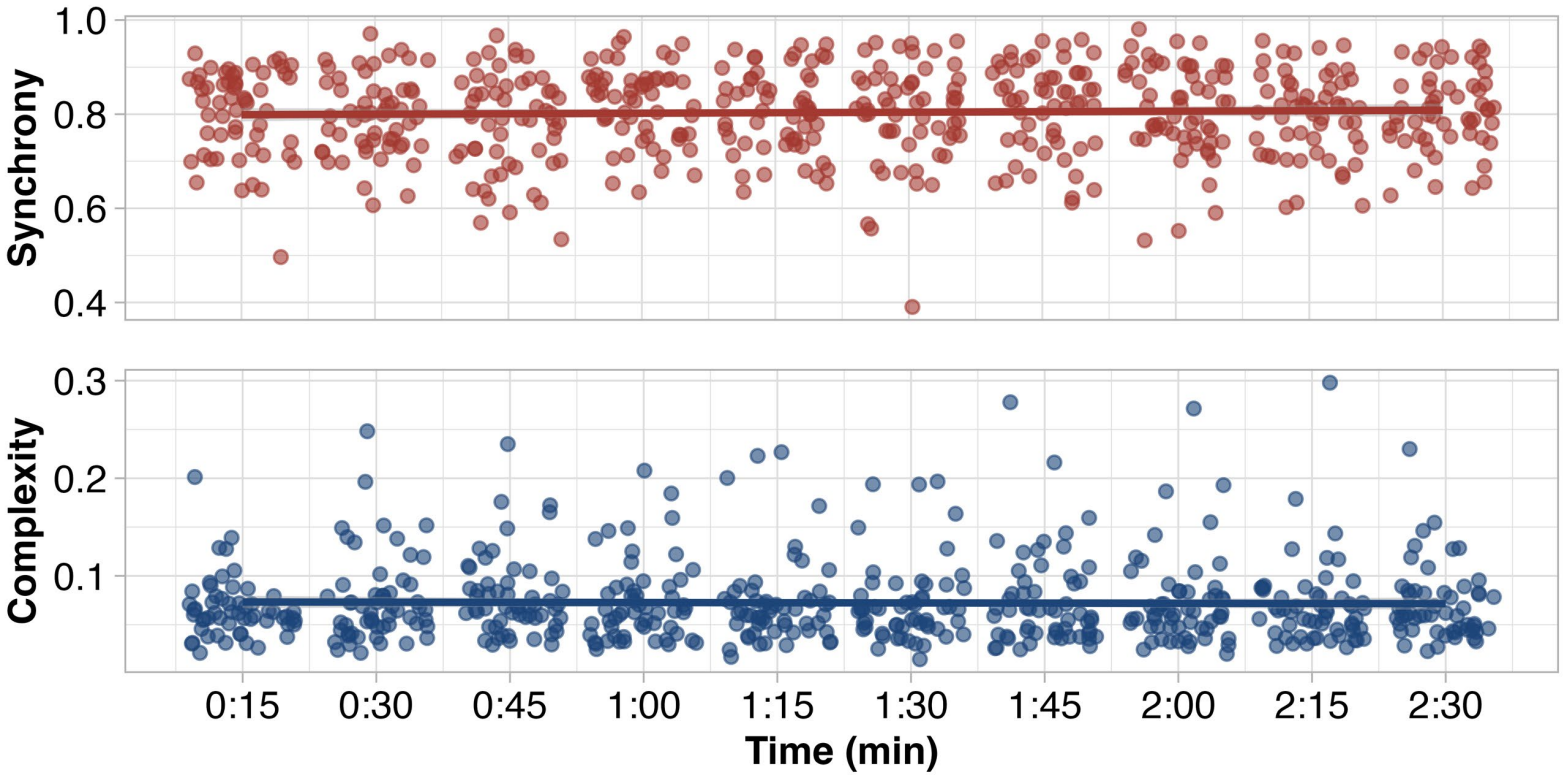
Development of movement synchrony and over the course of the 2.5-min mirror game, in discrete 15-s windows.

Our final hypothesis was that movement synchrony and complexity may change over the course of a prolonged mirror game, and that dyadic body competence and/or perception may interact with this change. We found no evidence for any of these simple or interactional relationships, as adding *Time* as a parameter in our models did not improve the explanation of the variance in the data substantially (Figure 3).

**Figure 4:**
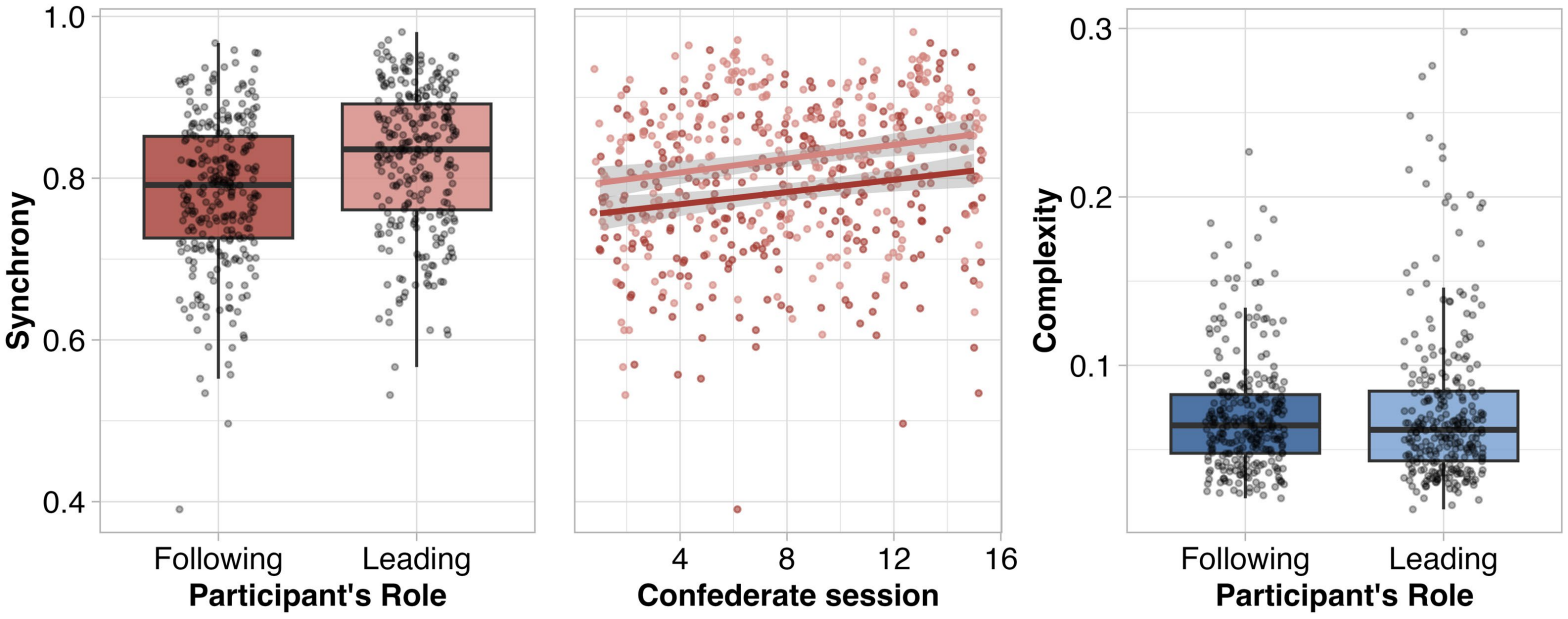
Left: Movement synchrony when the participant takes the role of the mirror-game leader and follower. Middle: Relationship between level of synchrony and confederate session (a proxy for increasing expertise) with participant as leader (lighter hue) and follower (darker hue). Right: Movement complexity when the participant takes the role of the mirror-game leader and follower.

In addition to the preregistered analyses above, we also explored the extent to which the mirror-game expertise of the mirror-game follower, i.e., the person copying their partner’s movements, may influence the overall level of movement synchrony or complexity (Figure 4). We compared levels of movement synchrony and complexity when the mirror game was led by the participant (for whom the mirror game is novel) vs. the confederate (who has played the mirror game multiple times). We found that movement synchrony was greater when the confederate matched the participants’ movements as opposed to when the participant matched the confederates’ movements [participant following - participant leading: *ß* = −0.04, SE = 0.01, 95% CI (−0.05, −0.03), *p* < 0.001]. That is, movement synchrony was greater when the expert followed the non-expert’s movements. Further exploratory analyses revealed expertise accrued by confederates across sessions contributed significantly to explaining variance, when treated as a simple fixed effect in combination with leading or following role. Confederate session was positively associated with level of synchrony [*ß* = 0.01, SE = 0.01, CI (0.00, 0.01), *p* = 0.031; middle pannel of Figure 4]. For movement complexity, the participant’s role did not explain substantial variance in the data and did not meet our criteria for inclusion in the model. As a result, we have no evidence for a relationship between movement complexity and the participant’s role in the mirror game.

## 4 Discussion

In this study, we took a dyad-centered approach to understanding the relationship between embodiment and the synchrony and complexity of movements that emerge while a dyad strives to maintain movement synchrony. We found that greater shared embodiment, in terms of dyadic body competence scores, was associated with greater movement synchrony, but not movement complexity. Dyadic body perception did not contribute to explaining inter-dyad differences in movement synchrony or complexity. We also observed that both movement synchrony and complexity remained stable over the course of the mirror game and found no evidence that the time course of movement synchrony and complexity was related to dyadic measures of embodiment. We remind readers that these exploratory analyses of a secondary dataset are to be interpreted with caution. Our motivation for these analyses was to stimulate hypothesis generation, and we recommend that the findings be subjected to further empirical examination with larger sample sizes.

### 4.1 Dyadic body competence and mirroring expertise enhance movement synchrony

We found that dyads with higher body competence scores showed greater movement synchrony during the mirror game. This finding aligns with existing research demonstrating that expert dancers and improvisors, whose livelihoods or hobbies involve creative but controlled body movements, achieve greater movement synchrony than non-experts (Noy et al., 2011; Honisch, 2012; Hart et al., 2014; Kirsch et al., 2016). Thus, our findings are consistent with existing evidence that embodiment is important for achieving synchrony. Moreover, the positive association between dyadic body competence scores and movement synchrony highlights two important phenomena. First, embodiment gained through the general public’s (in this case, Australian undergraduate students’) everyday activities and hobbies also contributes to the ability to achieve movement synchrony with a stranger. Second, a high degree of movement synchrony is more likely when both members of a dyad have strong positive beliefs about their body’s ability to complete physical tasks.

We also observed that movement synchrony was greater when the experimental confederate was the follower rather than the leader. We initially interpreted this to mean that the level of movement synchrony is impacted differently by a dyad member’s bodily experience with the mirror game, depending on whether they are leading or following. We are thankful to a reviewer for raising the possibility that confederates’ knowledge of the study’s hypotheses, the fact that the movement synchrony would be evaluated, and the social benefits of movement synchrony described in the literature, may have engendered a different form of motivation than that experienced by participants. Indeed, it appears (middle pannel of Figure 4), that synchrony increased alongside the number of sessions completed by each experimental confederate, suggesting that confederates’ expertise does indeed play a role in the level of synchrony. However, the overall difference in the level of synchrony when the participant was leading and following is consistent across confederate sessions (i.e., no interaction), suggesting that the differing forms of motivation experienced by participants and experimental confederates might underpin the difference in synchrony between participant and confederate-led movements.

Considering the influence of expertise on movement synchrony first, our findings add to existing evidence from Hart et al. (2014) that dyads comprising two expert improvisors, relative to two novice improvisors, can achieve greater movement synchrony in the mirror game. That is, greater embodiment is particularly beneficial for supporting the perception–action processing involved in matching another person’s movements in time and space (Honisch et al., 2016). With greater embodiment (i.e., body competence or experience mirroring), movement synchrony may emerge more fluidly in interactions with others. This proposition aligns with evidence that embodiment, gained through physical experience, may enable more fluent simulation of observed movements, which in turn is associated with greater activation of sensorimotor brain regions while observing familiar movements (Cross et al., 2006; Jola et al., 2012; Kirsch et al., 2016). Second, we turn to the probable influence of differing motivations on movement synchrony in synchronizing activities, such as the mirror game. This aligns with existing findings, suggesting individuals’ motivations (i.e., to bond with others) are predictive of movement synchrony levels (Miles et al., 2010; Lumsden et al., 2012; Fujiwara et al., 2020). Moreover, the perspective that motivations shape movement synchrony highlights a potential weakness of experimental confederates for studying movement synchrony. Considered differently, the present work provides preliminary evidence suggesting that expertise with synchrony and motivation to maintain synchrony should be considered when designing studies or interventions involving synchronizing activities.

Moreover, our findings have useful implications for contexts where movement synchrony is operationalized to improve social connections or cognition (e.g., Ramseyer and Tschacher, 2011; Tunçgenç and Cohen, 2016; Keisari et al., 2022; Moffat et al., 2024). Greater synchrony is likely to ensue when a trained or experienced individual takes the role of the follower. We also propose that experience can be accrued, as our experimental confederates were not ‘experienced’ at playing the mirror game prior to helping with the data collection, and the experience gained through data collection was sufficient for them to improve their perception-action correspondences in such a way that led to greater synchrony.

### 4.2 Measures of embodiment do not predict complexity of dyadic movements

We did not find movement complexity to be modulated by any measures of embodiment (body competence/perception) or bodily experience with the mirror game. We interpret this as evidence that successful synchronization relies more on the follower’s perception and matching of movements than the leader’s adjustment of movement complexity. In other words, we found no evidence that dyadic or individual levels of embodiment were associated with behaviors like making simpler or more predictable movements to help the follower, or more complex, less predictable, movements as a challenge to the follower. This observation is reinforced by our findings that movement complexity remained stable over the course of the mirror game.

We anticipated synchrony might increase over the course of the mirror game as participants become entrained with each other’s movements and that this may co-occur with less complex movement that could be more easily predicted by the follower. We also evaluated an alternative prediction based on Ravreby et al.’s (2022) report that novelty and complexity of mirror game movements facilitate greater interpersonal liking: we anticipated that dyads might increase the complexity of their movements over time, thereby reducing synchrony, as they may subconsciously seek more positive social experiences as the mirror game progressed. A linear increase in novelty, but not complexity is indeed visible in Ravreby et al.’s (2022) data visualizations. While our findings regarding the stability of movement synchrony and complexity are aligned with Ravreby’s visualization, it is possible that a non-linear approach to quantifying changes in movement synchrony or complexity over time may offer more nuanced insights. With respect to synchrony, it is possible that the overall level of synchrony resulting from the mirror game is too high with too little variance between dyads from the onset of the game. In other words, our data may show a type of ceiling effect for synchrony. Another consideration is that a larger sample size would be beneficial for assessing changes over time, as the effect size of such changes might be smaller than detectable with the current sample (see Data analysis section for note on statistical power).

### 4.3 Dyadic perspectives for illuminating dyadic interactions

In this study, we explicitly considered dyadic scores related to embodiment (i.e., body competence and perception) as opposed to individual scores. While information about individuals can shed light on how individuals might behave, information about dyads offers more detailed insight into dyadic behaviors that emerge in interactive social contexts (Arellano-Véliz et al., 2023). Our findings add to the relatively sparse landscape of studies describing how dyad-level traits, experiences and skills influence the emergence of interpersonal synchrony by showing that greater dyadic embodiment enhances the level of synchrony that a dyad can achieve. We do not wish to neglect the wealth of research examining individual traits in relation to social interactions, as opposed to dyads’ traits (e.g., Lakens and Stel, 2011; Miles et al., 2011; Ragert et al., 2013; Tschacher et al., 2014, 2018; Brambilla et al., 2016; Cheng et al., 2017; Lahnakoski et al., 2020; Stupacher et al., 2020; Zimmermann et al., 2021; Glass and Yuill, 2023). Instead, we strive to highlight the value of examining dyads’ behavior using dyadic measures. Future work could, for example, further explore additional dyadic measures that pertain to embodiment, such as experience with the arts (Casale et al., 2023), or even dyadic disembodiment traits (Ciaunica et al., 2023).

Another potential avenue for future research is related to the increasing prevalence of social interactions of non-human agents (i.e., chatbots, social robots) in everyday life. Non-human agents’ embodiment, specifically the kinematics of their interactions with humans, is central to their acceptance by users (Lorenz et al., 2016; Nalepka et al., 2019; Rinott and Tractinsky, 2022). A deeper understanding of the emergence and maintenance of movement synchrony within human–robot dyads may help enhance perceptions, acceptance and then ensuing interactions (Henschel and Cross, 2020). Existing evidence suggests that people prefer social robots with personalities resembling their own (Aly and Tapus, 2013), emphasizing how dyadic approaches are equally promising for understanding interactions with non-human agents.

## 5 Conclusion

We set out to explore the influence of dyadic indices of embodiment (body competence and perception) on dyads’ abilities to achieve and maintain movement synchrony, and the complexity of the movements they made during the mirror game. The data revealed that higher body competence scores and mirror-game expertise, but not body perception scores, allowed dyads to achieve greater movement synchrony. Movement complexity during the mirror game was not influenced by any measure investigated here. Moreover, the data suggest that movement synchrony and complexity remain relatively stable across mirror game rounds. We conclude by emphasizing the value of dyadic approaches for uncovering the dynamics that modulate dyadic body movements. Dyadic approaches stand to enable the design of more effective movement synchrony interventions to promote social and cognitive behaviors.

## Data availability statement

Publicly available datasets were analyzed in this study. This data can be found at: https://osf.io/gd5nb/.

## Ethics statement

The studies involving humans were approved by Macquarie University Human Research Ethics Committee. The studies were conducted in accordance with the local legislation and institutional requirements. The participants provided their written informed consent to participate in this study.

## Author contributions

RM: Conceptualization, Data curation, Formal analysis, Investigation, Methodology, Project administration, Supervision, Writing – original draft, Writing – review & editing. LR: Conceptualization, Formal analysis, Investigation, Methodology, Visualization, Writing – original draft, Writing – review & editing. CC: Writing – original draft, Writing – review & editing. EC: Conceptualization, Funding acquisition, Project administration, Resources, Supervision, Writing – original draft, Writing – review & editing.

## Footnotes

^1^https://osf.io/6rc3g
